# Improved Quality of Life in A Case of Cerebral Palsy after Bone Marrow Mononuclear Cell Transplantation

**DOI:** 10.22074/cellj.2016.3754

**Published:** 2015-07-11

**Authors:** Alok Sharma, Hemangi Sane, Pooja Kulkarni, Myola D’sa, Nandini Gokulchandran, Prerna Badhe

**Affiliations:** 1 Department of Medical Services and Clinical Research, NeuroGen Brain and Spine Institute, StemAsia Hospital and Research Centre, Navi Mumbai, India; 2 Department of Research and Development, NeuroGen Brain and Spine Institute, StemAsia Hospital and Research Centre, Navi Mumbai, India; 3 Department of Neurorehabilitation, NeuroGen Brain and Spine Institute, StemAsia Hospital and Research Centre, Navi Mumbai, India

**Keywords:** Cerebral Palsy, Cell Therapy, Autologous, Bone Marrow, Mononuclear Cells

## Abstract

Cerebral palsy (CP) is a non progressive, demyelinating disorder that affects a child’s development and posture and may be associated with sensation, cognition, communication and perception abnormalities. In CP, cerebral white matter is injured resulting in the loss of oligodendrocytes. This causes damage to the myelin and disruption of nerve conduction. Cell therapy is being explored as an alternate therapeutic strategy as there is no treatment currently available for CP. To study the benefits of this treatment we have administered autologous bone marrow mononuclear cells (BMMNCs) to a 12-year-old CP case. He was clinically re-evaluated after six months and found to demonstrate positive clinical and functional outcomes. His trunk strength, upper limb control, hand functions, walking stability, balance, posture and coordination improved. His ability to perform activities of daily living improved. On repeating the Functional Independence Measure (FIM), the score increased from 90 to 113. A repeat positron emission tomography-computed tomography (PET-CT) scan of the brain six months after intervention showed progression of the mean standard deviation values towards normalization which correlated to the functional changes. At one year, all clinical improvements have remained. This indicated that cell transplantation may improve quality of life and have a potential for treatment of CP.

## Introduction

Cerebral palsy (CP) describes a group of disorders of development and posture that cause activity limitations. These are attributed to non-progressive disturbances that occur in the developing fetal or infant brain. The motor disorders often occur along with difficulty in communication, perception, disturbed sensation, cognition, and/or seizures (1). CP is the most common physical disability in childhood, occurring in 2.0 to 2.5 per 1000 live births (2). Risk factors highly depend on the timing of the occurrence-prenatal, perinatal, and postnatal (3). Spasticity, contractures, drooling, osteopenia, osteoporosis and difficulty in performing all activities of daily living are the major complications involved in CP (4). Management strategies involve physiotherapy, occupational therapy and surgical and medical interventions (5). Management is not curative; but optimum care can improve the quality of life of these children and their families (6).

Cell transplantation is being actively explored as a treatment alternative for neurological disorders such as CP, brain injuries, stroke, and spinal cord injuries as stem cells have shown regenerative and reparative potential. Stem cells promote angiogenesis, neurogenesis, reduce inflammation and increase oxygen supply to the brain (7-10).

In our case study, we evaluated the clinical and functional outcome after intrathecal transplantation of autologous bone marrow mononuclear cells (BMMNCs) in a 12-year-old boy diagnosed with CP.

## Case report

A 12-year-old boy with spastic diplegic CP was born at 33 weeks of gestation by C-section delivery. He was the second child among of twins with low birth weights and poor Apgar scores. He cried immediately after birth and was kept on a ventilator for a week and in the incubator for three weeks. Gradually, the parents noticed that his milestones were delayed compared to his twin brother. A delay in neck holding was noted at the age of six months so he was investigated and later diagnosed to have CP. He did not have convulsions. Since the age of nine months, he underwent continuous rehabilitation therapy.

On neurological examination, he was hypertonic and hypereflexic. All sensations were intact. The voluntary control in the lower extremities was poor with presence of minimal spasticity. Fine motor activity of the upper extremity was found to be fair. He walked using elbow crutches with a crouch gait indoors and used a wheelchair outdoors. Bilateral flexion attitude of both knees was present but fully stretchable. The feet had rocker bottom deformity. Strength of the abdominal and back muscles was reduced and his trunk balance was poor which attributed to his crouch posture.

Functionally, he required assistance in most activities of daily living. He had normal bowel and bladder control. On the Functional Independence Measure (FIM) he scored 90. On the Gross Motor Function Classification System (GMFCS) he was level III. Cognition was normal with age appropriate comprehension and expression. Speech was unclear and slurred with a nasal twang. Oromotor skills were adequate. Magnetic resonance imaging (MRI) brain revealed diffuse ill-defined peritrigonal hyper intensity bilaterally with paucity of parietal white matter and prominent trigones of both lateral ventricles. The imaging features were consistent with periventricular leucomalacia. Electroencephalography (EEG) did not show epileptiform activity. The positron emission tomography-computed tomography (PET-CT) scan of the central region, cerebellum, vermis, supplementary motor areas and paracentral lobule showed abnormality.

### Procedure

Patient selection and protocol design was performed according to the inclusion criterion of the World Medical Association Helsinki Declaration (11). The protocol was reviewed and approved by the Ethics Committee and Institutional Committee for Stem Cell Research and Therapy (ICSCRT).

The patient’s parents were informed about the procedure and a duly filled informed consent was obtained. Granulocyte colony-stimulating factor (G-CSF, 300 μg) injections were administered subcutaneously, 48 hours and 24 hours before the intervention. Pre-intervention assessment included extensive evaluation by a team of medical and rehabilitation experts. Detailed neurological and functional evaluation was documented.

Bone marrow (100 ml) was aspirated from the iliac bone under local anesthesia using a bone marrow aspiration needle and collected in heparinized tubes. The mononuclear cells (MNCs) were separated by the density gradient method and checked for cluster of differentiation 34^+^ (CD34^+^) and viability. CD34^+^ counting was performed by fluorescence activated cell sorting (FACS). The viability was 98%. Approximately 33×10e^6^ MNCs, diluted in the patient’s own CSF, were immediately injected intrathecally in the lumbar vertebral (L4-L5) space. We intravenously administered 1 gm Solumedrol in 500 ml Ringer Lactate solution at the time of transplant. Following transplantation, he underwent intensive neurorehabilitation that included physiotherapy, occupational therapy and speech therapy as a part of the treatment program. The patient was placed on a personalized exercise program that emphasized techniques to facilitate mobility and multiplication of the injected stem cells thereby giving enhanced results.

**Fig.1:**
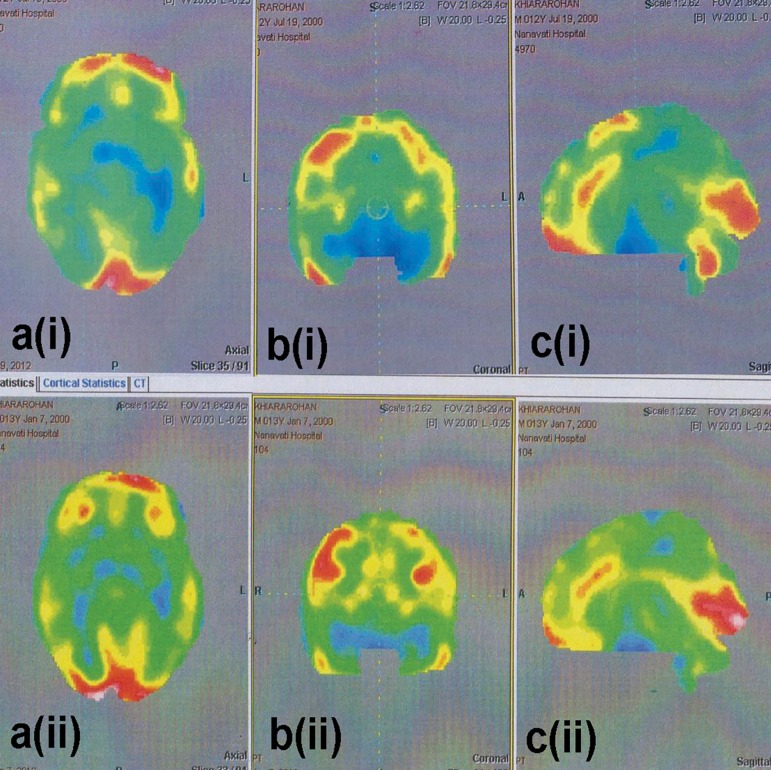
PET-CT scan images obtained before intervention [a(i), b(i), c(i)] and six months after intervention [a(ii), b(ii), c(ii)]. In a(i), b(i), and c(i), blue areas indicate hypometabolism. In a(ii), b(ii) and c(ii), these areas have decreased and turned green which is indicative of normal metabolism. PET-CT: Positron emission tomography- computed tomography.

A week after the therapy, he began to show improvements. Trunk strength and upper limb control improved. The patient was walking with bilateral push knee splints and elbow crutches. Crouch gait reduced. He could wear his t- shirt on his own, independently and eat by himself. At the six month follow up, he kneel-walked for at least 45 minutes. Trunk balance and control had further improved. Transfers such as bed, sitting and getting up from the floor were performed in a controlled manner and easier. Posture was more erect. Walking stability had improved. In activities of daily living, he could eat with better coordination, dress himself with minimal assistance, and achieved independent toileting activities. Hand functions improved, due to which his writing speed increased with a better handwriting. On repeating the FIM, the score increased from 90 to 113, with improvements in upper and lower body dressing, toileting, bladder and bowel management and transfers. On repeating the PET-CT scan of the brain six months after the intervention, the mean standard deviation values of the central region, cerebellum, vermis, supplementary motor areas and paracentral lobule progressed towards normalization (Fig.1). It was observed that the functions of these areas of the brain had improved (Table 1). All clinical improvements have been sustained until the end of one year. The patient will be followed every six months thereafter to further assess his progress.

**Table 1:** Areas of the brain that show a positive shift in mean SD values and functional improvements corresponding to these areas


Areas where the mean SD valuesshifted towards normalization	Mean SDvalue before therapy	Mean SDvalue after therapy	Improved functions

Supplementary motor areas	2.8(left)	2.1(left)	Motor functions, voluntary motor control, motorcoordination, locomotion, bimanual coordination, control of sequence of movements andpostural stability
	2.1(right)	1.9(right)	
Cerebellum areas 4-5	-1.8 (left)	-0.4 (left)	Trunk balance, walking balance, coordination,fine motor activities
Cerebellum area 6	-3.4 (left)	-2.5 (left)	Trunk balance, walking balance, coordination,fine motor activities
Mesial temporal lobe	-6.3 (left)	-3.8 (left)	Memory
Olfactory cortex	-1.2 (left)	-0.3 (left)	Sense of smell
	-2.4 (right)	- 0.6 (right)	
Vermis 4-5	2.8	1.1	Maintaining balance, body posture and movement
Vermis 6	3.3	2.3	Maintaining balance, body posture and movement
Vermis 7	2.6	1.2	Maintaining balance, body posture and movement
Vermis 8	9.4	4.1	Maintaining balance, body posture and movement
Vermis 9	8.4	1.1	Maintaining balance, body posture and movement
Central region	3.9(left)	3.8(left)	Coordination
Superior frontal gyrus	3.6(left)	2.9(left)	Complex motor functions
Thalamus	-2.6 (left)	-1.4 (left)	Motor control processing
	-2.0 (right)	-1.7 (right)	


SD; Standard deviation.

## Discussion

CP is a non-progressive, demyelinating disease. Cell transplantation is being explored as a novel, promising treatment for CP (12). Not many results from clinical trials have been published to date (13). A variety of cells have been used for treatment such as olfactory ensheathing cells, umbilical cord blood cells, bone marrow cells, and adipose tissue cells (14, 15). We have administered autologous BMMNCs to a 12-year-old CP patient. Bone marrow derived cells are a preferred cell source due to their capability for self-renewal, proliferation and pluripotent differentiation. They are easily obtainable and do not cause an immune rejection after transplantation; hence they do not present any ethical controversies. These cells are safe and no case of tumorogenicity has been reported until now. BMMNCs are a mixture of various hematopoietic and non-hematopoietic cells (mesenchymal cells). Studies have shown that the mixture of cells is more beneficial than its sub-fractions (16). Bone marrow mesenchymal cells (BMMSCs) comprise a significant fraction of these cells. They are known to exhibit neural phenotypes and differentiate into neuron-like cells (17). BMMNCs as a whole are promising candidates for cell therapy as they help in tissue repair and immune process regulation (18).

In our case study, administration of these cells showed a positive functional outcome. The case showed a significant change in his daily activities and improvement in quality of life, which was reflected by an increased FIM score. These changes were attributed to several mechanisms of the stem cells.

Injected BMMNCs survive and migrate to the site of injury, after which they restore damaged neural cells (19). Some cells in the MNCs perform repair through cell replacement while other cells secrete cytokines which indirectly assist repair and regeneration (20). These cells release neurotrophic factors such as brain-derived neurotrophic factor (BDNF), nerve growth factor (NGF) and vascular endothelial growth factor (VEGF), which are considered key factors that promote brain function recovery (21). In animal models of brain injury treated with bone marrow derived stem cells (BMSCs), a higher concentration of these neurotrophic factors has been observed compared to the control group. VEGF also induced angiogenesis, improving blood circulation in the injured regions (22, 23). These factors also stimulated the endogenous repair process of the brain.

Cerebral white matter injury is a common feature in CP that results in loss of oligodendrocytes. This causes damage to the myelin and disruption of nerve conduction (24). BMMNCs have a potential to differentiate into oligogodendrocytes and astroglial cells which carry out the repair process by remyelinating axons (25).

In a recent study carried out by Chen et al. (26), 60 CP cases received autologous neural stem cell (NSC)-like cells derived from bone marrow. They found that this was a safe, effective treatment for motor deficits in CP. In our previous case studies on CP and CP with mental retardation (MR), we demonstrated significant improvement in brain metabolism as shown by the PET-CT scan performed after cell therapy (27, 28). Similar results were also reported in studies carried out on brain stroke and spinal cord injury patients. The patients improved functionally and these improvements correlated with changes in PET-CT scans in brain stroke and MRI and American Spinal Injury Association (ASIA) in spinal cord injury (29, 30).

This case study reinforced the previous findings of using a PET-CT scan as a monitoring tool for effects of cellular therapy in CP. The comparison of standard deviation (SD) values before and after intervention, showed a trend toward normalization. The functional improvement demonstrated by the patient corresponded to the areas of the brain that showed evidence of change in the SD values. This indirectly indicates restoration of neuronal functions in the areas shown in table 1. Future cases should study serial PET-CT scans at longer intervals which may provide further insight about the long-term effect of cellular therapy. Larger randomized clinical trials should be required to assess the safety and benefit of this therapy. The correlating clinical improvements with functional benefits and improved quality of life direct us to explore cellular therapy as a potential modality of treatment for CP.
